# Posterior Urethral Valves

**DOI:** 10.1100/tsw.2009.127

**Published:** 2009-10-14

**Authors:** Steve J. Hodges, Bhavin Patel, Gordon McLorie, Anthony Atala

**Affiliations:** Department of Urology, Wake Forest University School of Medicine, Winston-Salem, NC, USA

**Keywords:** posterior urethral valves, hydronephrosis

## Abstract

The most common cause of lower urinary tract obstruction in male infants is posterior urethral valves. Although the incidence has remained stable, the neonatal mortality for this disorder has improved due to early diagnosis and intensive neonatal care, thanks in part to the widespread use of prenatal ultrasound evaluations. In fact, the most common reason for the diagnosis of posterior urethral valves presently is the evaluation of infants for prenatal hydronephrosis. Since these children are often diagnosed early, the urethral obstruction can be alleviated rapidly through catheter insertion and eventual surgery, and their metabolic derangements can be normalized without delay, avoiding preventable infant mortality. Of the children that survive, however, early diagnosis has not had much effect on their long-term prognosis, as 30% still develop renal insufficiency before adolescence. A better understanding of the exact cause of the congenital obstruction of the male posterior urethra, prevention of postnatal bladder and renal injury, and the development of safe methods to treat urethral obstruction prenatally (and thereby avoiding the bladder and renal damage due to obstructive uropathy) are the goals for the care of children with posterior urethral valves[1].

Posterior urethral valves (PUV) are a congenital obstruction of the posterior urethra. As this disorder develops early in gestation, the bladder and upper urinary tract are exposed to elevated pressure throughout development, leading to far-reaching changes to bladder function and renal injury. Attempts to describe the origins of this disorder definitively, and therefore to develop methods for prevention, have been as frustrating as the attempts to alleviate the bladder obstruction during gestation. At the present time, the best that modern medicine has to offer for these children is close urologic care following delivery from a full gestation with maximization of bladder and renal function for the benefit of the child's long-term health[2].

## INCIDENCE

Obstruction of the fetal lower urinary tract is a rare disorder, affecting 2.2 per 10,000 births. PUV, while being the most common cause of lower urinary tract obstruction in males, are a rare disorder, affecting only 1 in 4000–7500 infants, and there is a disproportionately elevated incidence in African-Americans and children with Down's syndrome. Despite their rarity, PUV present such a severe insult to the upper urinary tract that they account for almost 17% of children with end-stage renal failure[3,4].

## EMBRYOLOGY AND CLASSIFICATION

The study of the embryologic origin and classification of PUV has led to a myriad of theories, with varying types and amounts of experimental and anatomic evidence. The male urethra is known to complete development at week 14 of gestation. The valves form prior to this time, producing some level of obstruction once urine production has begun for the remainder of fetal development. Valves were first described by Morgagni in 1717 and were described again by Lagenback in 1802 from the examination of autopsy specimens. The first scientific description of valves, their embryology, and their role in urinary tract pathology was presented by Tolmatschew in 1870. Perhaps the most famous description of valves came from Young in 1919, at which point the well-known PUV classification method was described[5].

According to the Young classification, there are three types of PUV, with Type I being by far the most commonly encountered. Type I (95%) represents sail-like folds from the verumontanum distally along the urethra. Type II valves likely have only historical significance and are not considered to be a clinical entity, but rather hypertrophied urethral folds. Type III (5%) valves represent a cannulated septum thought by some to represent an incomplete dissolution of the urogenital membrane.

Since that original classification, the description of PUV embryology has teetered between claims of abnormal integration of the Wolffian ducts into the urethra and exaggerations of normal folds seen in normal urethral development[5].

Interesting recent work on the topic claims that Type I and II valves represent the same entity, and are actually a COPUM (congenitally obstructing posterior urethral membrane) whose appearance may vary depending on the instrumentation or imaging studies performed. The term "Cobb's collar" has been applied to a distinct congenital urethral stricture occurring distal to an external urethral sphincter. COPUM appears to be a result of a persistent urogenital membrane, although the embryology of PUV is far from clear and an area fertile for research[5].

All theories of valve development point to an isolated event, although Wolffian duct abnormalities can have profound effects on the development of the genitourinary tract. Typically, valves are not found in syndromic conditions, but are usually an isolated disorder. Regardless, they can be associated with many other congenital urologic anomalies, such as hyposadias, prune belly syndrome, ureteropelvic junction obstruction, and crossed renal ectopia[3].

## PRESENTATION AND DIAGNOSIS

The most typical presentation for PUV in this day and age is detection of hydronephrosis during routine prenatal ultrasound (US), with subsequent definitive diagnosis following birth. In fact, 50–70% are diagnosed in the first year of life, with 25–50% seen neonatally. It is quite surprising that despite the widespread use of antenatal sonography, a good number of children do not present until well after birth[1,2].

The second trimester US that is now routine in obstetrical care and in cases of PUV commonly demonstrates bilateral hydroureteronephrosis with a thick walled bladder and keyhole sign in the bladder neck of males. This disorder may account for up to 10% of all prenatally detected hydronephrosis[1,2,3].

Cases of PUV that are not detected via prenatal US may often present after birth with findings such as poor urinary stream (although most children with valves may appear to void normally on simple examination of the urinary stream, and this is not a sign of the degree of obstruction), urinary tract infections, and failure to thrive. Older children may present with diurnal enuresis, infections, and severe voiding complaints, such as dribbling and retention, or hematuria[1,2].

The tools used to diagnose PUV in children include renal US to evaluate for the degree of hydronephrosis, bladder wall thickening, and the general health of the kidneys (Fig. 1). A reassuring sign in terms of renal function is good corticomedullary differentiation, while increased renal echogenicity and subcortical cysts are poor prognostic signs for renal function. A thick walled bladder on US may signify a poorly compliant bladder that, as is well understood, can lead to upper tract damage at filling pressures above 40 cm $H_2O$. Low compliance is not the only factor of thick walled bladder to affect renal function adversely, however, as these thick bladders also tend to obstruct the ureters physically as they fill[2,4,6].

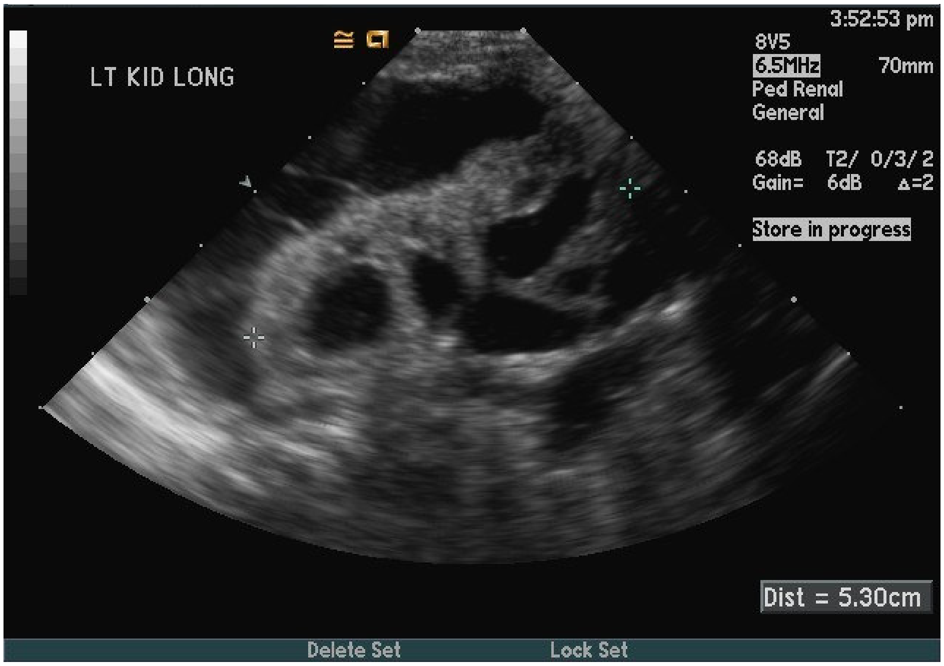

**FIGURE 1.** A renal US demonstrating hydronephrosis and a perinephric urinoma as a result of PUV.

The renal US, while an important part of the evaluation of a child with PUV, cannot make the definitive diagnosis. A voiding cystourethrogram (VCUG) is the only direct means of diagnosing valves, short of cystoscopic evaluation. In cases of PUV, a VCUG will demonstrate a dilated posterior urethra, trabeculated bladder, often vesicoureteral reflux, and often the valve leaflets themselves (Fig. 2). It is crucial to have a voiding view of the urethra with the catheter removed in order to make a complete evaluation of the urethra and to document all cases of valves[4].

## TREATMENT

The management of PUV consists of relieving the obstruction and pressure on the urinary tract, with care to maintain normal bladder and renal function for as long as possible. This has taken the form of catheter drainage of the bladder at birth, with close monitoring of serum electrolytes and renal function, and antibiotics to prevent urinary tract infections. Historically, children who did not improve clinically with bladder drainage (i.e., persistent hydronephrosis and elevated creatinine) would be subject to high urinary diversion (above the level of the bladder), with the belief that perhaps the thick bladder wall and altered anatomy prevented efficient drainage from a simple bladder catheter. Those who do improve with catheter

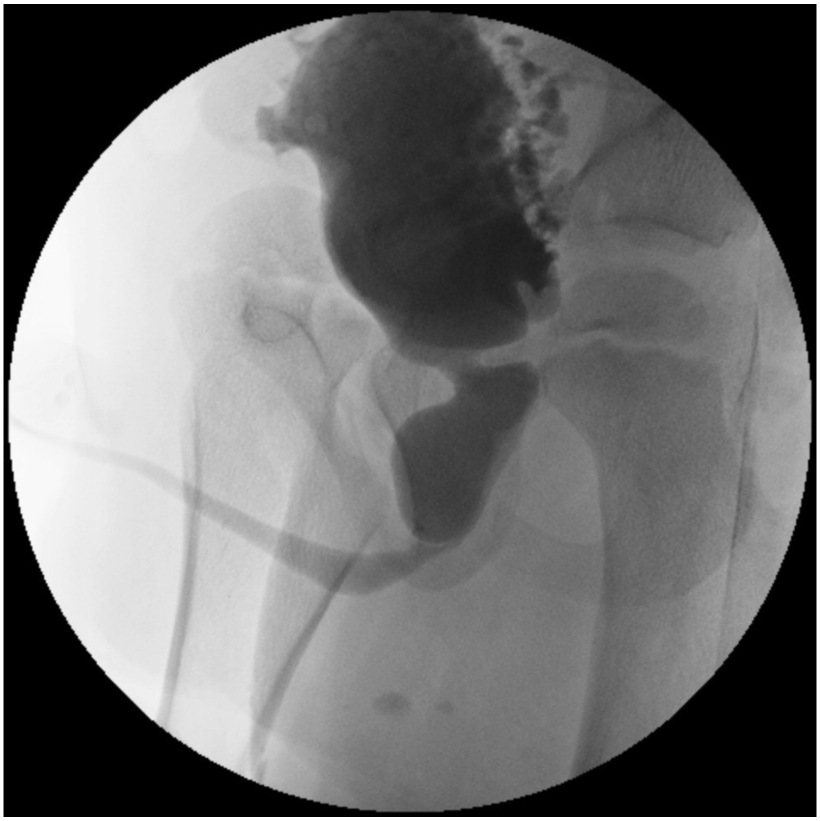

**FIGURE 2**. A voiding cystourethrogram demonstrating the trabeculated bladder, dilated posterior urethra, and valve leaflets typical of PUV.

drainage undergo endoscopic ablation of the valves, unless they are too small (under 2000 g), at which point vesicostomy can alleviate the obstruction until the children are large enough for definitive treatment. There may also be an advantage to vesicostomy in cases of severe reflux, as bladder pressure is reduced maximally following this procedure[3,7].

The routine use of high urinary diversion has fallen out of favor, however, as evidence suggested that bladder cycling is essential to normal bladder function in life[8]. This concept is debatable, however, with conflicting studies showing an actual long-term benefit to defunctionalization of the bladder in terms of functional capacity and compliance[6]. Regardless, the standard of care now continues to be catheter drainage until stable, followed by transurethral ablation of the valves using the small resectoscopes now available to urologists. Due to the improvement of these devices, most children can be ablated shortly after birth, with vesicostomies only required if the urethra will not allow for the passage of the resectoscope[7,9].

In reviewing the literature regarding therapy for PUV, there is good evidence to support any of the modern options of therapy. The earlier we are able to alleviate any obstruction or strain on the urinary tract, the better. However, the obstruction in PUV is an early development in gestation, initiated in the first trimester; therefore, any rush to treatment should be tempered by a long-term view of the problem. Although antenatal treatment is still experimental, and has been hampered by complications (including preterm labor), the future of valve therapy may well lie in fetal surgery, as the equipment and techniques used to treat fetal urinary tract obstruction evolve. It is safe to say, however, that at the current state of affairs, there is no need to seek fetal surgical options unless the mother is at one of the small number of medical centers actively investigating these techniques. In fact, early delivery options are even discouraged unless the fetal demise will result from inactivity.

In terms of upper tract diversion, a masterful review on the topic has been authored by Dr. Glassberg[6], who eloquently described the evidence behind all the therapy options. Glassberg's classification of ureters into grade I (unobstructed with empty and full bladder), II (unobstructed with empty, but obstructed with full bladder), and III (obstructed with full and empty bladder) demonstrated that very few cases of valve bladder actually have obstructed ureters, dictating that upper tract diversions are rarely necessary. In fact, in Glassberg's initial studies, no ureters were classified as type III and most were type II. However, historically, in cases when upper tract diversion was performed, those children did no better than those with lower tract diversions. Those opposed to upper tract diversions used this as evidence that there is no need to perform these diversions, as the end result is the same, while proponents of upper tract diversions felt these results provided some support for this practice, as these children were the most severely ill in all cases (yet had similar outcomes to children with much less ever conditions, presumably due to the improved drainage of upper tract diversion)[6,7].

In summary, the best practice guidelines appear to be to begin with catheter drainage and plan endoscopic valve ablation when the child is medically stable, and to follow the classic stepladder approach. If catheter drainage improves hydronephrosis and creatinine, then valve ablation is all that is necessary. If the hydronephrosis improves, but renal function deteriorates, this points to dysplastic kidneys that would not be helped by upper tract diversion. If, however, hydronephrosis and renal function worsens, an upper tract diversion may be warranted[7,9,10,11].

## ANTENATAL INTERVENTION

There has also been a push for the eminently logical approach of alleviating the urethral obstruction prenatally, to allow for normal renal and bladder development *in utero*. Unfortunately, this complex process has been hampered by failed procedures, preterm deliveries, fetal demise, and minimal improvement in outcomes. Indeed, it appears as though one of the important lessons that has been learned from studying PUV is that the damage done to the infant's urinary tract is an early process that begins at the initiation of urine production, and any attempts to change the anatomy or physiology of the disorder either prenatally or using extensive diversions of the urinary stream are all acts of futility, as the die (so to speak) has already been cast. Indeed, the evidence for renal dysplasia in cases of PUV due to abnormal budding of the mesonephric duct provide further proof that the fate of many of these children is decided at an early stage in development. In fact, the onset of a prenatal diagnosis of PUV with US has not changed the rate at which these children develop renal insufficiency[5,12,13].

Therefore, all things being equal, optimal management for the child with PUV includes treatment at birth and no sooner. If it were possible, however, to alleviate the urethral obstruction safely and restore normal bladder cycling to the fetus, the benefits would obviously be profound. Currently, the main indication for prenatal intervention is if amniotic fluid levels are decreasing during the crucial time of lung development (18–24 weeks). In these instances, efforts to increase amniotic fluid can help to prevent pulmonary dysplasia[1,3,4].

In certain cases, when low amniotic fluid levels threaten fetal survival later in gestation, early delivery is often supported by the obstetricians. In these unfortunate situations, the morbidity due to PUV is often compounded by pulmonary complications due to poor lung development and early delivery[1].

In a handful of medical centers, efforts are being undertaken to actually alleviate the urethral obstruction in the fetus. These treatment options take the form of percutaneous fetal cystoscopy and valve ablation, vesicoamniotic shunting, and even open fetal surgery. These highly complicated procedures are only undertaken when renal function is adequate and there is hope for benefit. Fetal urine samples are often tested and the renal prognosis is considered good if the fetal urinary sample demonstrates Na < 100 meq/l, Cl < 90 meq/l, osmolality < 200 mosm/l, and beta2 microglobulin < 6 mg/l. The benefits of these procedures are currently being studied in a randomized trial, the PLUTO (Percutaneous shunting in Lower Urinary Tract Obstruction) trial, which will hopefully provide some guidance regarding this highly controversial topic[4,14,15].

## LONG-TERM MANAGEMENT

If the fate of the renal function in PUV is often set at birth, assuming optimal management from that point on, then more useful tools than aggressive treatment of valves would be more accurate prognostic factors for future renal function. For example, echogenic kidneys, especially with multiple cysts, signify poor future renal function. These somewhat subjective parameters can be supported by serum measurement of creatinine, which has been demonstrated to predict the likelihood of renal failure if above 1 mg/dl at 1 year of age[16].

Other positive predictors of renal function are glomerular filtration rate at diagnosis, ascites, diverticula, and unilateral reflux or VURD syndrome (vesicoureteral reflux and renal dysplasia), as these are all popoff valves that decrease the pressure faced by the kidneys during gestation (Fig. 3). Negative predictive factors (predictive of renal failure) include elevated BUN, late intervention, reflux nephropathy, growth retardation, bilateral reflux at time of diagnosis, presentation before 1 year of age (the earlier the diagnosis, the worse the prognosis), distal renal tubular acidosis, and incontinence by day at age 5 years. Hyperfiltration injury may also develop in cases of PUV. Proteinuria in infancy carries a worse prognosis and as this predicts that the child will develop hyperfiltration as it grows, it may lead to focal segmental glomerular sclerosis and renal failure[2,17,18,19,20].

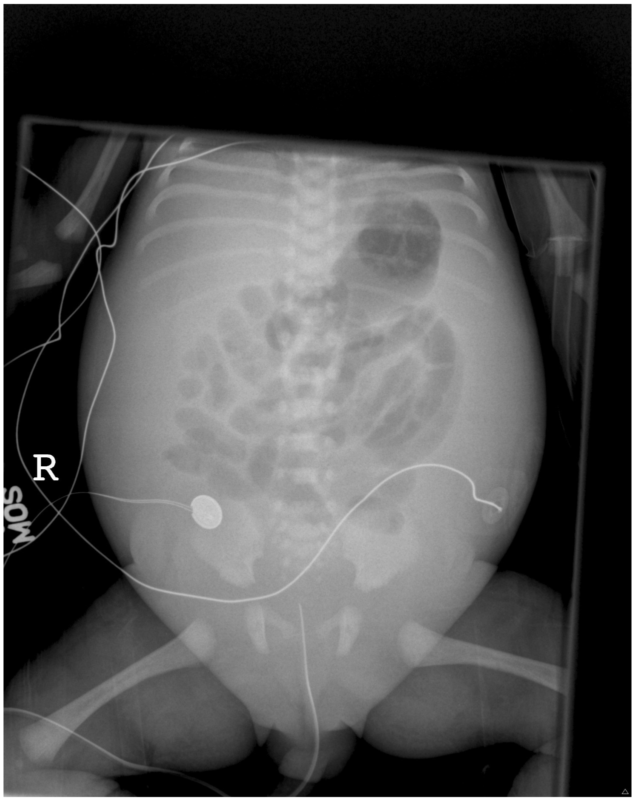

**FIGURE 3.** An abdominal radiograph demonstrating abdominal urinary ascites as a result of the elevated pressure in the urinary tract and calyceal rupture.

There is debate as well as to the causes of renal function in patients with PUV. If renal failure occurred early in life, then it was likely due to renal dysplasia that was congenital. Renal failure that develops later in life may be due to poor bladder function leading to further renal injury, which may be preventable with appropriate monitoring and treatment[1,21].

Close follow-up is needed after valve ablation to ensure proper bladder function in the children. For example, persistent severe reflux that prevents efficient bladder emptying, poorly functioning bladders, or UVJ (ureterovesical junction) obstruction due to detrusor hypertrophy may require an alternate procedure (such as urinary diversions) to maximize emptying and protect renal function. In addition, antibiotic prophylaxis is crucial to avoid urinary tract infections, in order to prevent any further damage to already tenuous kidneys[1].

There have been many classifications of bladder function in patients with PUV, as well as the development of the concept of the "valve bladder"[22]. The description of the valve bladder syndrome by Peters is useful in following these patients long term[23]. Of course, all children with PUV are different and do not necessarily follow any schedule in terms of deteriorating bladder function, but it is clear that different patterns of bladder pathology are seen specifically in children with PUV. Peters divided these into three groups: hyper-reflexic, small noncompliant, and myogenic failure[23]. These categories tend to proceed in order, with children beginning with hyper-reflexic bladders (that may respond to anticholinergic therapy) and then often descending into small noncompliant bladders, that may need medical therapy and catheterization, or even augmentation. Interestingly, overnight catheter drainage has proven to be protective in some patients, by reducing the strain of high urine output on the urinary tract, with resultant normal bladder function in these children following transplant[24]. The final stage for many of these children is myogenic failure, with the bladder losing contractile ability, in which case catheterization is necessary for emptying[6].

In addition to the concerns of the valve bladder syndrome on renal function, the valve bladder may also lead to incontinence, with as many as 17–70% of children complaining of wetting at advanced ages. The two main causes of this incontinence are bladder dysfunction and sphincter incompetence. These children require management with timed/double voiding, anticholinergics, and clean intermittent catheterization. In cases where conservative measures are not successful in protecting renal function, enterocystoplasty is required[2,3].

While the valve bladder syndrome was historically thought to arise from the antenatal damage done to the bladder from the valvular obstruction, bladder damage may progress following valve ablation. There may be evidence of persistent bladder neck obstruction in children with valves postablation, and alpha adrenergic blockade may be beneficial in these instances. In addition, the kidneys in these children often have a concentration defect, producing large amounts of dilute urine. The large volume of urine may lead to persistent urinary tract dilation and (if the child has poor sensation and poor bladder emptying) a chronically distended bladder, which leads to further bladder injury[1,2,3]. Close follow-up of bladder function with US studies and urodynamics is essential in order to maintain maximal bladder function. In fact, the damage done by polyuria may be alleviated by initiating an overnight catheter, so that the bladder is given a long period of rest during a time that it would otherwise likely be distended[24].

In the long term, many children with PUV develop end-stage renal disease and require renal transplantation. Historically, they had very poor results with renal transplants, as compared to age-matched controls, however, our expanding understanding of the valve bladder and techniques to improve bladder function have equalized results in these patients[2].

## CONCLUSION

There is still much to be learned about PUV, and the embryology and pathology of urethral development remains a field of active investigation. Simple questions regarding the origins of the disorder and its classification remain unanswered. It is not uncommon for the research of a rare clinical entity to raise more questions than answers and, in fact, it is a testament to the extensive research that has been performed over the past century that the traditional explanations and classification of PUV have been questioned, altered, and clarified. Hopefully, the future will continue to produce new revelations regarding this intriguing disorder, with an eye toward the prevention or successful antenatal treatment of this devastating disorder.
